# Which is better to use “body weight” or “standard liver weight”, for predicting small‐for‐size graft syndrome after living donor liver transplantation?

**DOI:** 10.1002/ags3.12412

**Published:** 2020-12-11

**Authors:** Takeo Toshima, Tomoharu Yoshizumi, Tomonari Shimagaki, Huanlin Wang, Takeshi Kurihara, Yoshihiro Nagao, Shinji Itoh, Noboru Harada, Masaki Mori

**Affiliations:** ^1^ Department of Surgery and Science Graduate School of Medical Sciences Kyushu University Fukuoka Japan

**Keywords:** graft weight per standard liver weight, graft‐to‐recipient weight ratio, liver transplantation, living donor liver transplantation, small‐for‐size graft syndrome

## Abstract

**Aim:**

Little evidence about whether to apply graft‐to‐recipient body weight ratio (GRWR) or graft weight to standard liver weight (GW/SLW) for graft selection has been published. The aim of the present study was to clarify the importance of the correct use of GRWR and GW/SLW for selecting graft according to the recipients’ physique in living donor liver transplantation (LDLT).

**Methods:**

Data were collected for 694 recipients who underwent LDLT between 1997 and 2020.

**Results:**

One of the marginal grafts meeting GW/SLW ≥ 35% but GRWR < 0.7% has been used in more recipients with men and higher body mass index (BMI), and the other meeting GRWR ≥ 0.7% but GW/SLW < 35% has been used in more recipients with women with lower BMI. In the cohort of BMI > 30 kg/m^2^, the recipients with GRWR < 0.7% had a significantly higher incidence of small‐for‐size graft syndrome (SFSS) compared to those with GRWR ≥ 0.7% (*P* = 0.008, 46.2% vs 5.9%), and using the cutoff of GW/SLW < 35% could not differentiate. In contrast, in the cohort of BMI ≤ 30 kg/m^2^, the recipients with GW/SLW < 35% also had a significantly higher incidence of SFSS (*P* = 0.013, 16.9% vs 9.4%). Multivariate analysis showed that GRWR < 0.7% [odds ratio (OR) 14.145, *P* = 0.048] was the independent risk factor for SFSS in obese recipients, and GW/SLW < 35% [OR 2.685, *P* = 0.002] was the independent risk factor in non‐obese recipients.

**Conclusion:**

Proper use of the formulas for calculating GRWR and GW/SLW in choosing graft according to recipient BMI is important, not only to meet metabolic demand for avoiding SFSS but also to ameliorate donor shortages.

## Graphical abstract

This is the first report to demonstrate the importance of proper use of GRWR and GW/SLW for graft selection according to recipient physique in the setting of LDLT. Taken together, the way to calculate the GW cutoff values should be changed, according to the recipient BMI value to prevent postoperative SFSS after LDLT.

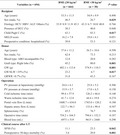

**Abbreviations**
BMI: body mass index
CT: computed tomography
GRWR: graft‐to‐recipient weight ratio
GW: graft weight
LDLT: living donor liver transplantation
POD: postoperative day
SFSS: small‐for‐size syndrome
SLW: standard liver weight

## 1 INTRODUCTION

Living donor liver transplantation is being increasingly carried out worldwide to address the shortage of donor organs.^1^ Surgical techniques result in a partial graft that has a reduced overall parenchymal mass compared with whole organ allograft.^2^ Smaller grafts incapable of meeting all of the metabolic, synthetic, and hemodynamic demands of recipients have been implicated as a cause of early allograft dysfunction.^3^ The constellation of persistent ascites, cholestasis, and coagulopathy in the setting of a reduced‐size graft without an obvious technical cause has been termed SFSS.^4^, ^5^

Smaller‐size grafts can enhance donor safety and expand donor availability; however, they also cause SFSS, which has high mortality and morbidity rates.^6^, ^7^ There are two ways to calculate the required graft volume for the recipient: (a) GRWR (%, GW/recipient body weight × 100), and (b) GW to SLW (%, GW/SLW).^8^, ^9^ At most high‐volume centers for LDLT, the minimum acceptable GRWR is 0.6%–0.8% or GW/SLW 30%–40%.^10^, ^11^, ^12^, ^13^, ^14^, ^15^, ^16^, ^17^ However, some patients have SFSS even with adequate GW. Furthermore, it is unclear whether GRWR or GW/SLW is more accurate in predicting sufficient GW and is effective in preventing SFSS after LDLT. This discrepancy may highlight the importance of other factors responsible for graft dysfunction in the small‐for‐size graft. Local hemodynamic effects such as portal hyperperfusion, impaired venous outflow, and recipient disease severity also contribute to graft injury and therefore influence critical graft size.^18^, ^19^

To the best of our knowledge, little evidence about whether to apply GRWR or GW/SLW for graft selection has been published. Furthermore, there are no reports that these two parameters are used properly according to the recipients’ physique.

Therefore, the aim of the present study was to clarify the importance of properly using GRWR and GW/SLW for graft selection according to the recipients’ physique in the setting of LDLT.

## 2 METHODS

### 2.1 Patient characteristics

Consecutive adult recipients who underwent LDLT at Kyushu University Hospital (Fukuoka, Japan) from May 1997 through May 2020 were enrolled in the study and data were collected. Indications for LDLT (n = 694) were liver cirrhosis resulting from hepatitis C (n = 238), cholestatic cirrhosis (n = 135), acute liver failure (n = 82), hepatitis B (n = 61), alcohol abuse (n = 60), non‐alcoholic steatohepatitis (n = 37), autoimmune hepatitis (n = 20), and other conditions (n = 61).

Living donor liver transplantations were carried out after obtaining full informed consent from all patients and approval by the Liver Transplantation Committee of Kyushu University. The study protocol was carried out in accordance with The Code of Ethics of the World Medical Association (Declaration of Helsinki) and the Kyushu University Hospital Institutional Review Board (No. 2019‐186).

### 2.2 Graft selection

Donors were required to be spouses or within the third degree of consanguinity with the recipients as well as between the ages of 20 and 65 years. For a donor who was not within the third degree of consanguinity, individual approval was obtained from the Kyushu University Hospital Ethics Committee.^20^ We used three‐dimensional computed tomography (CT) for preoperative volumetric analysis and delineation of vascular anatomy. SLW of recipients was calculated according to the formula: 706.2 × body surface area + 2.4.^21^ The two formulas of GRWR and GW/SLW are the formulas for predicting SFSS and measuring the indication for LT “before LDLT”; therefore, BW‐related values, such as BMI and body surface area, were calculated by using wet BW which includes ascites volume. GW was predicted by computed tomographic volumetric analysis.

We have used only GW/SLW for the indication of LDLT, without GRWR; GW/SLW is above 35%.^19^, ^22^ The type of graft selected for the recipients was based on the preoperatively predicted GW/SLW.^10^ A left lobe graft with or without the caudate lobe was procured if the estimated GW/SLW was ≥35%. A right lobe graft was procured if the estimated GW/SLW using the extended left lobe with the caudate lobe was <35% and the preoperatively predicted remnant liver volume of the donor was ≥35%. A right posterior sector graft was considered when the remnant liver volume after right hepatectomy was <35%.^23^ When using a right lobe graft, the middle hepatic vein tributaries draining segment 5 and 8 veins (V5 and V8) and the inferior right hepatic vein were detected by preoperative three‐dimensional CT. Reconstruction of these veins was indicated when their diameter was ≥5 mm or their volume was ≥10% of the GW.^22^

Regarding portal flow modification, our basic strategy was to close any shunts as much as possible to prevent the phenomenon of portal steal and graft hypoperfusion. An exceptation is that when the portal pressure increased above 20 mm Hg by shunt clamping, we leave large shunts.^24^ The indications for simultaneous splenectomy are portal hypertension indicated by splenomegaly, huge portosystemic shunt, risky esophagogastric varices, portal pressure above 20 mm Hg after portal reperfusion, severe hypersplenism, and ABO blood type‐incompatible donor, and splenic artery aneurysms.^19^ The graft types included: left lobe with caudate lobe graft (n = 341), right lobe graft without the middle hepatic vein (n = 321), left lobe without caudate lobe graft (n = 16), right lobe graft with the middle hepatic vein (n = 3), and posterior segment graft (n = 13). For analysis, the actually procured GW was used, not the GW that was estimated preoperatively.

### 2.3 Postoperative management

The graft harvesting technique, recipient surgery, and recipient perioperative management, including immunosuppression regimens, have been previously described.^2^, ^7^ Immunosuppression was initiated with a protocol based on tacrolimus (Prograf; Astellas Pharma Inc., Tokyo, Japan) or cyclosporine A (Neoral; Novartis Pharma KK, Tokyo, Japan), with a steroid and/or mycophenolate mofetil (Chugai Pharmaceutical Co., Ltd, Tokyo, Japan).^5^, ^6^ The target trough concentration for tacrolimus was set at 10 ng/mL for 3 months after LDLT, followed by 5‐10 ng/mL thereafter. The target trough concentration for cyclosporine A was set at 250 ng/mL for 3 months after LDLT, followed by 150‐200 ng/mL thereafter. Methylprednisolone was initiated on the day of LDLT, and then tapered and converted to prednisolone 7 days after LDLT. Prednisolone treatment was tapered and discontinued 6 months after LDLT. Mycophenolate mofetil was used in 547 recipients (82.5%) and was started at 2000 mg/d on the next day after LDLT, and then tapered and discontinued until 6 months after LDLT. All recipients had monthly follow ups. The median follow‐up period was 2482 days; 719 days and 3996 days corresponded to the 25th and 75th percentiles, respectively.

### 2.4 Definition of SFSS

Small‐for‐size graft syndrome was defined as having both prolonged functional cholestasis and intractable ascites.^4^ Prolonged functional cholestasis was defined as total bilirubin > 10 mg/dL at postoperative day (POD) 14, without any other definitive causes for cholestasis. Ascites production was defined as the daily volume of ascites through indwelling drains. Intractable ascites was defined as a daily production of ascites of >1 L at POD 14 or >500 mL at POD 28.

### 2.5 Statistical analysis

All statistical analyses were carried out using JMP statistical software, version 15 (SAS Institute Inc., Cary, NC, USA) and R, version 3.2.1 (R Foundation for Statistical Computing, Vienna, Austria). Continuous variables were expressed as the mean ± standard deviation and compared using the nonparametric Wilcoxon test for independent samples. The chi‐squared test was used to compare categorical values. A logistic regression analysis was applied to the multivariate analyses.^5^, ^25^ Survival was calculated with the Kaplan‐Meier product‐limited method; differences in survival between the groups were compared with the log‐rank test. *P*‐values < 0.05 were considered statistically significant.

## 3 RESULTS

### 3.1 Correlation between GRWR and GW/SLW

**Figure 1:**
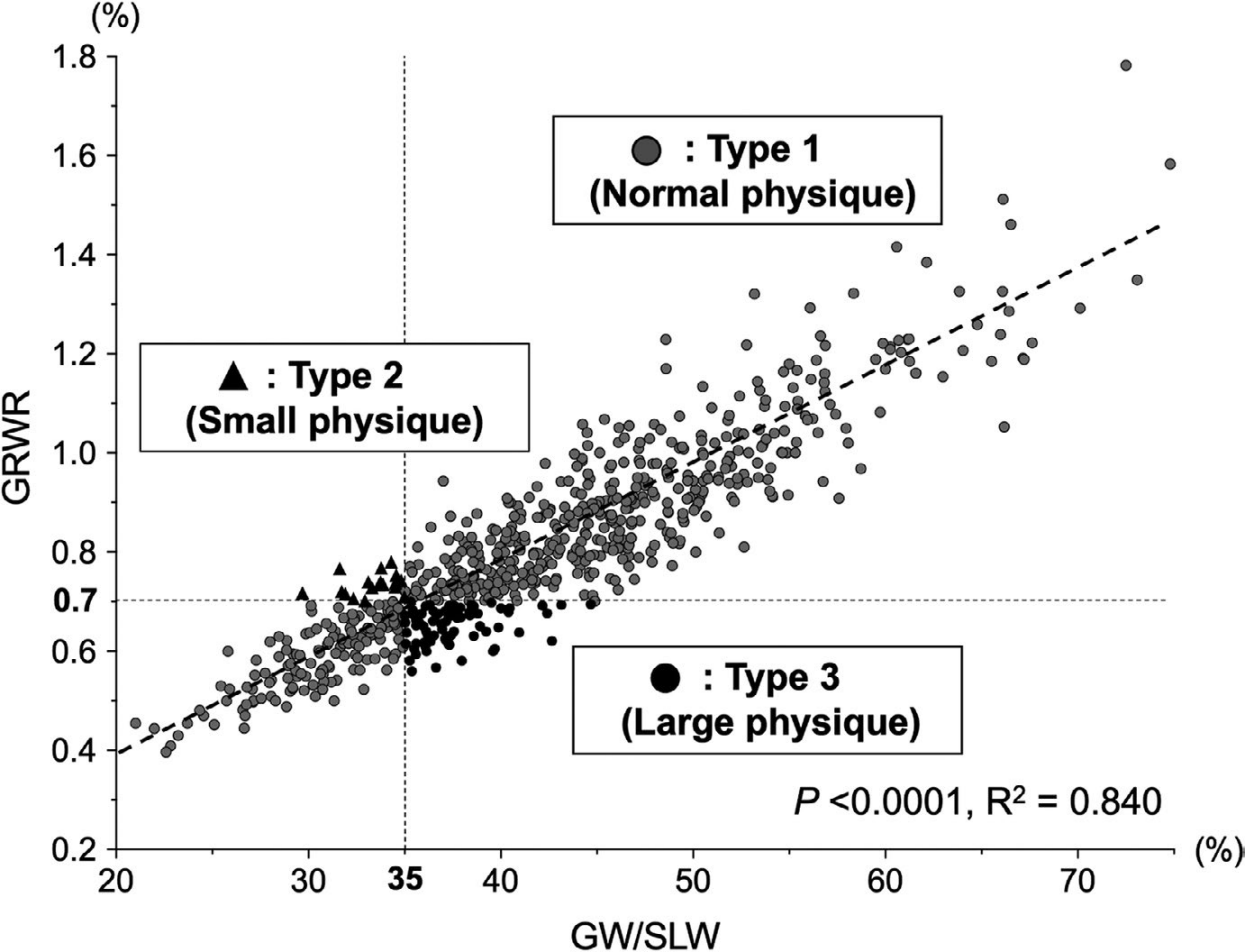
Graft‐to‐recipient body weight ratio (GRWR) and graft weight to standard liver weight (GW/SLW) were significantly correlated by using the actual GW in living donor liver transplantation (LDLT; n = 694). Recipients were divided into three physique groups by the cutoff values of GRWR 0.7% and GW/SLW 35%; type 1 (n = 595, the cohort with a consistent correlation between GRWR and GW/SLW values), type 2 (n = 18, the cohort of GRWR ≥ 0.7% but GW/SLW < 35%), and type 3 (n = 81, the cohort of GW/SLW ≥ 35% but GRWR < 0.7%). GW, graft weight; SLW, standard liver weight

**Figure 2:**
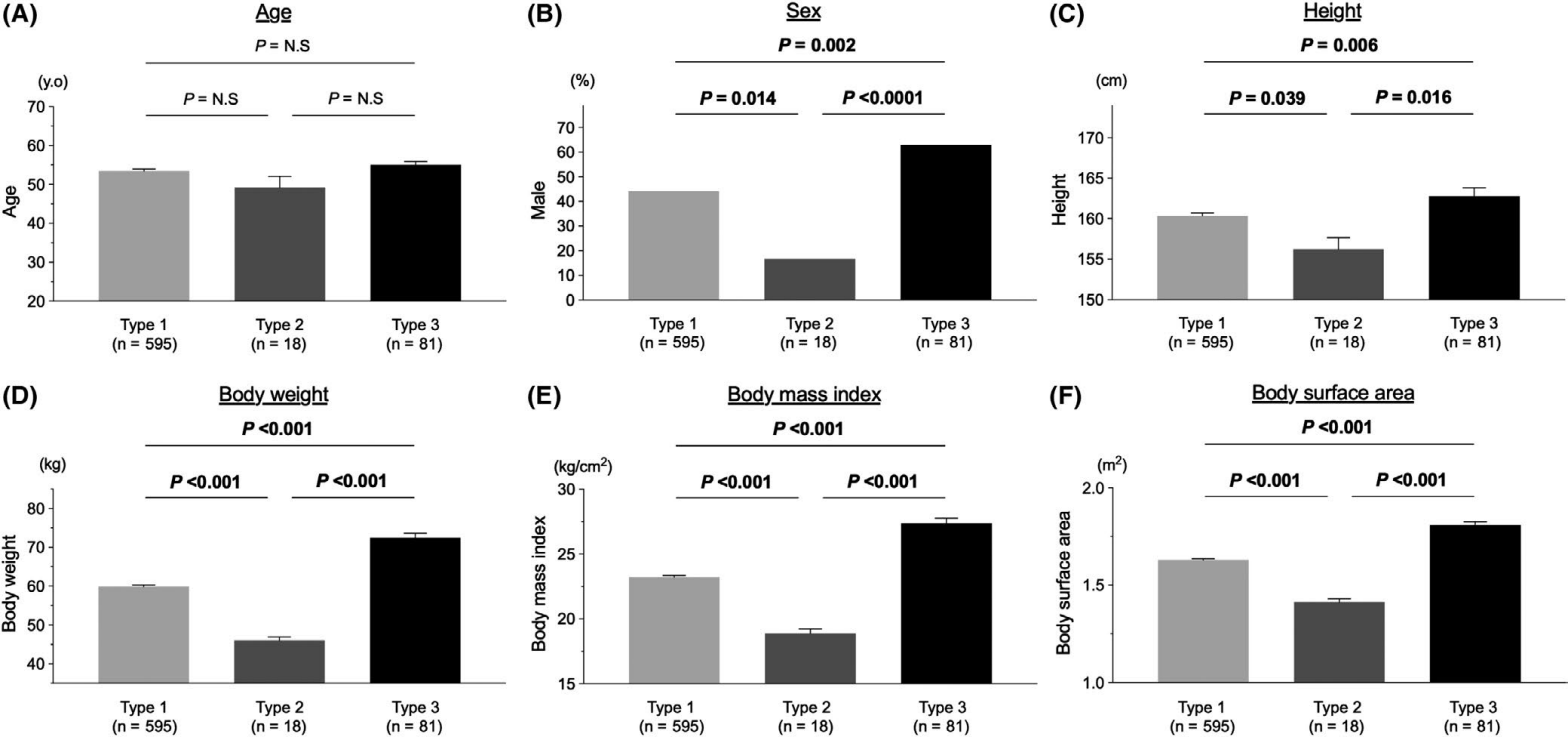
Differences in the characteristics of living donor liver transplant recipients by the three physique types defined in Figure 1 (n = 694). A, There was no significant difference in age among these three groups. B–F, Compared with type 1 and type 2, type 3 had significantly more men (B), taller height (C), and heavier weight (D). In addition, type 3 had significantly higher body mass index (E) and body surface area (F)

We measured the correlation between GRWR and GW/SLW in all 694 recipients by using the actual GW. GRWR and GW/SLW were significantly correlated with *R* ^2^ = 0.840 (*P* < 0.0001) (Figure 1). Regarding GW for LDLT, in many institutions, the accepted safe and ideal cutoff value for GRWR is 0.7% and for GW/SLW is 35%.^10^, ^11^, ^15^, ^26^ By using these cutoff values, we checked the difference in the correlation between these two calculated values (GRWR and GW/SLW). This study population (n = 694) was divided into three groups: type 1 (the cohort with a consistent correlation between GRWR and GW/SLW values), type 2 (the cohort of GRWR ≥ 0.7% but GW/SLW < 35%), type 3 (the cohort of GW/SLW ≥ 35% but GRWR < 0.7%). Next, the difference in the recipients’ characteristics differentiated by these three types of physiques was measured; the results are shown in Figure 2. There was no significant difference in age among these three groups. Compared with type 1 and type 2, type 3 had significantly higher numbers of men, higher height, and heavier weight. Type 3 also had significantly higher BMI and body surface area.

Collectively, these data demonstrated that the recipients can be divided into three population types depending on the physique; one of the marginal grafts meeting GW/SLW ≥ 35% but GRWR < 0.7% has been used in more male recipients and recipients with larger physique, and the other meeting GRWR ≥ 0.7% but GW/SLW < 35% has been used in more female recipients and recipients with smaller physique.

### 3.2 Difference between GW/SLW and GRWR for predicting SFSS according to physique

**Figure 3:**
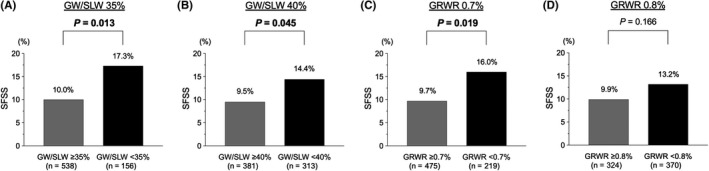
Differences in the incidence of small‐for‐size graft syndrome (SFSS) by each cutoff value of graft weight to standard liver weight (GW/SLW; 35% and 40%) and graft‐to‐recipient body weight ratio (GRWR; 0.7% and 0.8%) in the recipients who underwent living donor liver transplantation (LDLT; n = 694). A, Recipients with GW/SLW < 35% had a significantly higher incidence of SFSS compared with those with GW/SLW ≥ 35% (*P* = 0.013, 17.3% vs 10.0%). B, Recipients with GW/SLW < 40% had a significantly higher incidence of SFSS compared with those with GW/SLW ≥ 40% (*P* = 0.045, 14.4% vs 9.5%). C, Recipients with GRWR < 0.7% had a significantly higher incidence of SFSS compared with those with GRWR ≥ 0.7% (*P* = 0.019, 16.0% vs 9.7%). D, There was no significant difference in the SFSS incidence between the recipients with GRWR < 0.8% and those with GRWR ≥ 0.8%. GW, graft weight; SLW, standard liver weight

The incidence of SFSS was examined by each cutoff value of GW/SLW 35%‐40% or GRWR 0.7%‐0.8% (Figure 3). When the GW cutoff was set to GW/SLW 35%, the recipients whose GW was below the cutoff had a significantly higher incidence of SFSS than those above the cutoff level (*P* = 0.013, 17.3% vs 10.0%). Similar results were obtained for both GW cutoff as GW/SLW 40% (*P* = 0.045, 14.4% vs 9.5%) and GRWR 0.7% (*P* = 0.019, 16.0% vs 9.7%). There was no significant difference in the SFSS incidence between the recipients with GRWR < 0.8% and those with GRWR ≥ 0.8%.

Next, considering the fact that the physique was different in the recipients in whom the differences were between the GRWR and GW/SLW values, we focused on whether the optimal cutoff values of GW/SLW or GRWR could properly differentiate the incidence of SFSS by using the physique of the recipient. Because BMI was the factor that had the most difference between GW/SLW and GRWR, we set the cutoff as BMI 30 kg/m^2^, which is defined as obesity by the World Health Organization.^27^ Table 1 shows the characteristics of the recipients, donors, operation, and clinical course after liver transplantation according to the two groups: the recipients with BMI ≤ 30 kg/m^2^ (n = 664) and with BMI > 30% (n = 30). Compared with recipients with BMI ≤ 30 kg/m^2^, the obese group (BMI > 30 kg/m^2^) included fewer males (*P* = 0.029), more recipients with Child‐Pugh grade C (*P* = 0.017), more recipients with right lobe (*P* = 0.001), more recipients who had a higher incidence of SFSS (*P* = 0.042), and longer postoperative 30‐day mortality (*P* = 0.001). Regarding the GW, the obese group (BMI > 30 kg/m^2^) also had a significantly heavier GW (*P* = 0.001); however, they had a lower rate of GW/SLW < 35% (*P* = 0.017) and a similar rate of GRWR < 0.7%, compared with the recipients with BMI ≤ 30 kg/m^2^.

**Table 1:** Comparison of clinical characteristics between the patients with BMI ≤ 30 kg/m^2^ (n = 664) and BMI > 30 kg/m^2^ (n = 30)

Variables (n = 694)	BMI ≤ 30 kg/m^2^ (n = 664)	BMI > 30 kg/m^2^ (n = 30)	*P* value
Recipient			
Age (years)	53.5 ± 11.5	54.9 ± 9.9	0.498
Gender (male, %)	46.5	26.7	**0.029**
Etiology: HCV/ HBV/ ALF/ Others (%)	33.9/ 8.9/ 11.9/ 45.3	43.3/ 6.7/ 10.0/ 40.0	0.766
Presence of HCC (%)	38.3	40.0	0.848
Child‐Pugh C (%)	63.1	83.3	**0.017**
MELD score	16.2 ± 7.8	19.0 ± 6.1	0.051
Preoperative condition: hospitalized (%)	39.0	53.3	0.121
Donor			
Age (years)	37.6 ± 11.2	36.5 ± 10.6	0.598
Gender (male, %)	62.4	73.3	0.213
Blood type: ABO incompatible (%)	12.8	20.0	0.282
Graft type: Right lobe (%)	45.2	80.0	**0.001**
GW (g)	482.1 ± 113.6	574.9 ± 110.3	**0.001**
GW/SLW < 35% (%)	23.2	6.7	**0.017**
GRWR < 0.7% (%)	31.0	43.3	0.167
Operation			
PV pressure at laparotomy (mm Hg)	24.6 ± 6.1	25.8 ± 5.2	0.306
PV pressure at closure (mm Hg)	15.9 ± 3.7	17.0 ± 6.5	0.150
Cold ischemic time (min)	99.4 ± 57.9	126.2 ± 66.6	0.140
Warm ischemic time (min)	41.7 ± 12.8	44.4 ± 19.8	0.274
Portal vein flow (L/min)	1600.7 ± 634.0	1765.0 ± 120.2	0.180
Hepatic artery flow (L/min)	122.7 ± 94.3	133.4 ± 99.4	0.547
Splenectomy (%)	65.5	76.7	0.194
Operative time (min)	754.2 ± 164.5	794.6 ± 152.3	0.187
Blood loss (mL)	6973 ± 519	9615 ± 2440	0.290
Clinical course after LT			
SFSS (%)	11.1	23.3	**0.042**
Postoperative 30‐day mortality (%)	3.6	20.0	**0.001**

*Note*: *P* < 0.05 is shown in bold as statistical significant.

Abbreviations: ALF, acute liver failure; BMI, body mass index; GRWR, graft‐to‐recipient weight ratio; GW, graft weight; HBV, hepatitis B virus; HCC, hepatocellular carcinoma; HCV, hepatitis C virus; MELD, Model for End‐Stage Liver Disease; PV, portal vein; SFSS, small‐for‐size graft syndrome; SLW, standard liver weight.

**Figure 4:**
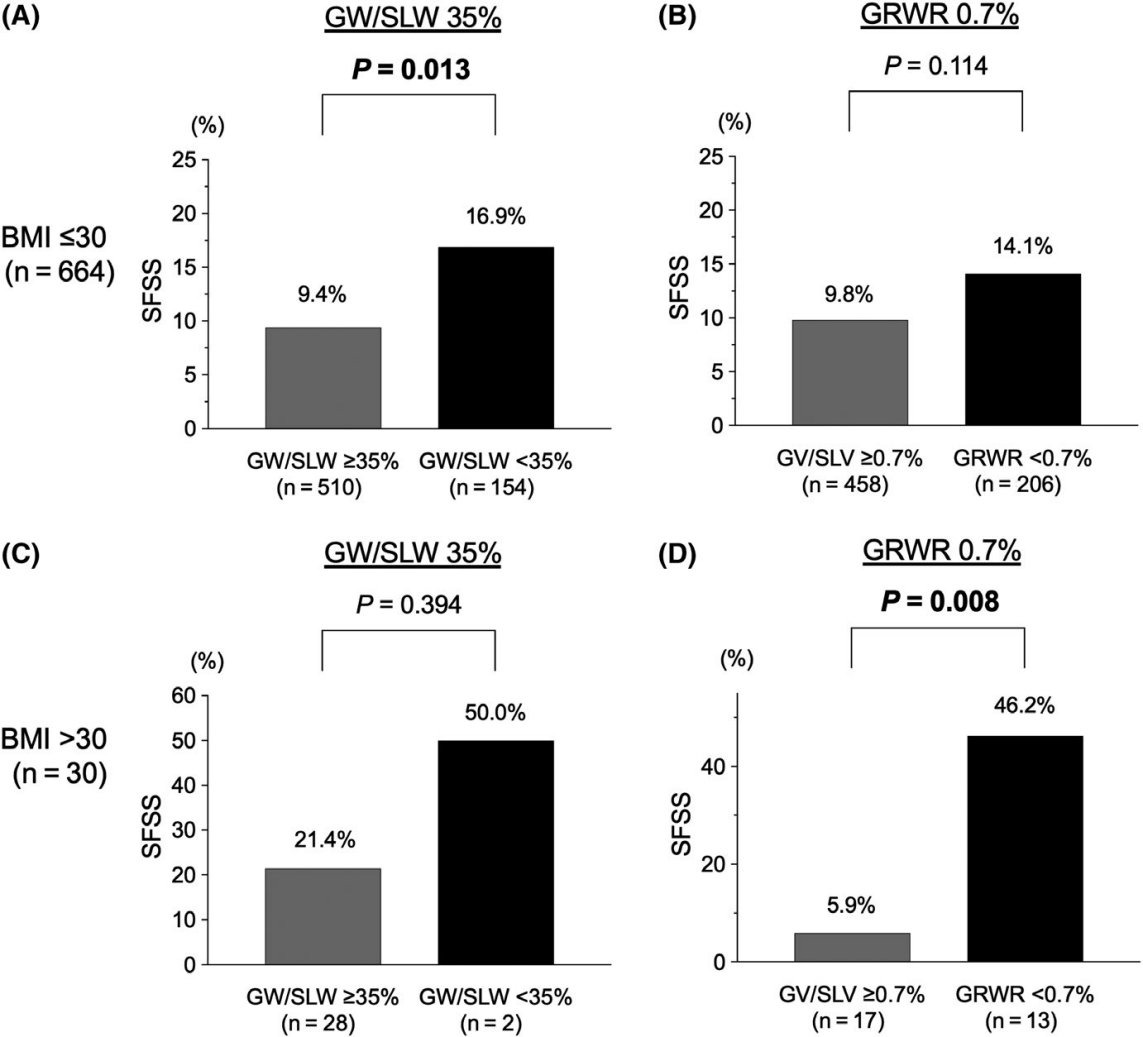
Differences in the incidence of small‐for‐size graft syndrome (SFSS) by the cutoff values of graft weight to standard liver weight (GW/SLW) 35% and graft‐to‐recipient body weight ratio (GRWR) 0.7% in recipients with body mass index (BMI) ≤ 30 mg/m^2^ (n = 664) and those with BMI > 30 mg/m^2^ (n = 30). (A,B) In the cohort of BMI ≤ 30 kg/m^2^, the recipients with GW/SLW < 35% had a significantly higher incidence of SFSS compared with those with GW/SLW ≥ 35% (*P* = 0.013, 16.9% vs 9.4%). There was no significant difference in the incidence of SFSS between the two when the cutoff values were set to GRWR 0.7%. (C,D) In the cohort of BMI > 30 kg/m^2^, the recipients with GRWR < 0.7% had a significantly higher incidence of SFSS compared with those with GRWR ≥ 0.7% (*P* = 0.008, 46.2% vs 5.9%). There was no significant difference in the incidence of SFSS between the two when the cutoff values were set to GW/SLW 35%. GW, graft weight; SLW, standard liver weight

In the cohort with BMI ≤ 30 kg/m^2^, the recipients with GW/SLW ≤ 35 kg/m^2^ had a significantly higher incidence of SFSS compared with those with GW/SLW > 35 kg/m^2^ (*P* = 0.013, 16.9% vs 9.4%) (Figure 4A); however, there was no significant difference between the two groups when the GW cutoff was set to GRWR of 0.7% (Figure 4B). In contrast, in the cohort of BMI > 30 kg/m^2^, the recipients with GRWR < 0.7% had a significantly higher incidence of SFSS compared with those with GRWR ≥ 0.7% (*P* = 0.008, 46.2% vs 5.9%); however, there was no significant difference between the two cohorts when the GW cutoff was set to a GW/SLW of 35% (Figure 4C,D).

Collectively, for predicting SFSS after LDLT, calculation of the GW cutoff value should be changed according to the physique of the recipient; the GW cutoff should be set as GRWR of 0.7% when the recipients have a BMI > 30 kg/m^2^ and GW/SLW of 35% when the recipients have a BMI ≤ 30 kg/m^2^.

### 3.3 Independent risk factors for SFSS

Univariate logistic regression analysis for postoperative SFSS in the recipients with BMI ≤ 30 kg/m^2^ showed the following risk factors: the presence of hepatocellular carcinoma (*P* = 0.037), Child‐Pugh grade C (*P* = 0.036), preoperative status at hospitalization (*P* = 0.003), donor age > 50 years (*P* = 0.024), left lobe graft (*P* = 0.038), GW/SLW < 35% (*P* = 0.011), portal vein pressure at closure > 20 mm Hg (*P* = 0.002), and the absence of splenectomy (*P* = 0.000) (Table 2). Next, multivariate logistic regression analysis using these eight factors showed that GW/SLW < 35% [odds ratio (OR) 2.685, 95% confidence interval (CI) 1.440‐5.008, *P* = 0.002] and four other factors: Child‐Pugh grade C (*P* = 0.001), donor age > 50 years (*P* = 0.002), portal vein pressure at closure > 20 mm Hg (*P* = 0.022), and the absence of splenectomy (*P* = 0.000) were independent risk factors for SFSS after LDLT in the recipients with BMI ≤ 30 kg/m^2^ (Table 2). In contrast, in the recipients with BMI > 30 kg/m^2^, multivariate logistic regression analysis using the two factors, which were revealed as the predictors for SFSS by univariate analysis, showed that both GRWR < 0.7% [OR 14.145, 95% CI 1.025‐195.263, *P* = 0.048] and PV pressure at closure > 20 mm Hg (*P* = 0.039) were independent risk factors for SFSS after LDLT in the recipients with BMI > 30 kg/m^2^ (Table 3).

**Table 2:** Risk factors for small‐for‐size graft syndrome in patients with BMI ≤ 30 kg/m^2^(n = 664)

Variables (BMI ≤ 30 kg/m^2^, n = 664)	Univariate analysis			Multivariate analysis		
	OR	95% CI	*P* value	OR	95% CI	*P* value
Recipient variables						
Age > 60 (years)	0.761	0.447‐1.297	0.316			
Gender (male)	0.760	0.465‐1.242	0.274			
Presence of HCC	0.563	0.329‐0.966	**0.037**	0.698	0.384‐1.269	0.238
Child‐Pugh C	1.797	1.040‐3.107	**0.036**	3.148	1.700‐5.828	**0.001**
MELD score > 20	1.454	0.852‐2.481	0.170			
Preoperative condition: hospitalized	2.120	1.302‐3.453	**0.003**	1.560	0.899‐2.707	0.114
Donor variables						
Age > 50 (years)	1.919	1.088‐3.386	**0.024**	2.753	1.458‐5.197	**0.002**
Gender (male)	1.130	0.682‐1.873	0.636			
Blood type: ABO incompatible	0.462	0.181‐1.180	0.107			
Graft type: Right lobe	0.584	0.352‐0.971	**0.038**	0.715	0.393‐1.301	0.272
GW/SLW < 35.0%	1.955	1.167‐3.275	**0.011**	2.685	1.440‐5.008	**0.002**
GRWR < 0.7%	1.504	0.913‐2.476	0.109			
Operation variables						
PV pressure at laparotomy > 20 mm Hg	0.939	0.461‐1.913	0.863			
PV pressure at closure > 20 mm Hg	2.759	1.433‐5.312	**0.002**	2.257	1.125‐4.527	**0.022**
Cold ischemic time (min)	0.998	0.993‐1.002	0.293			
Warm ischemic time (min)	0.995	0.976‐1.015	0.649			
Portal vein flow (L/min)	0.999	0.999‐1.001	0.214			
Hepatic artery flow (L/min)	0.997	0.994‐1.001	0.165			
Splenectomy	0.311	0.190‐0.511	**0.000**	0.254	0.145‐0.443	**0.000**

*Note*: *P* < 0.05 is shown in bold as statistical significant.

Abbreviations: ALF, acute liver failure; BMI, body mass index; CI, confidence interval; GRWR, graft‐to‐recipient weight ratio; GW, graft weight; HBV, hepatitis B virus; HCC, hepatocellular carcinoma; HCV, hepatitis C virus; MELD, Model for End‐Stage Liver Disease; OR, odds ratio; PV, portal vein; SFSS, small‐for‐size graft syndrome; SLW, standard liver weight.

**Table 3:** Risk factors for small‐for‐size graft syndrome in patients of BMI > 30 kg/m^2^ (n = 30)

Variables (BMI > 30 kg/m^2^, n = 30)	Univariate analysis			Multivariate analysis		
	OR	95% CI	*P* value	OR	95% CI	*P* value
Recipient variables						
Age > 60 (years)	0.381	0.038‐3.784	0.410			
Gender (male)	2.700	0.448‐16.255	0.278			
Presence of HCC	2.500	0.445‐14.037	0.298			
Child‐Pugh C	1.263	0.117‐13.591	0.847			
MELD score > 20	0.975	0.177‐5.385	0.977			
Preoperative condition: hospitalized	0.577	0.104‐3.186	0.528			
Donor variables						
Age > 50 (years)	7.875	0.980‐63.310	0.052			
Gender (male)	0.370	0.062‐2.230	0.278			
Blood type: ABO incompatible	0.600	0.058‐6.212	0.668			
Graft type: Right lobe	0.200	0.029‐1.374	0.102			
GW/SLW < 35.0%	3.667	0.199‐67.652	0.382			
GRWR < 0.7%	13.714	1.381‐136.212	**0.025**	14.145	1.025‐195.263	**0.048**
Operation variables						
PV pressure at laparotomy > 20 mm Hg	0.370	0.062‐2.230	0.278			
PV pressure at closure > 20 mm Hg	14.000	1.741‐112.551	**0.013**	14.501	1.148‐183.210	**0.039**
Cold ischemic time (min)	0.992	0.976‐1.009	0.349			
Warm ischemic time (min)	0.973	0.912‐1.039	0.413			
Portal vein flow (L/min)	0.999	0.997‐1.001	0.333			
Hepatic artery flow (L/min)	0.990	0.973‐1.007	0.256			
Splenectomy	0.281	0.563‐22.540	0.177			

*Note*: *P* < 0.05 is shown in bold as statistical significant.

Abbreviations: ALF, acute liver failure; BMI, body mass index; CI, confidence interval; GRWR, graft‐to‐recipient weight ratio; GW, graft weight; HBV, hepatitis B virus; HCC, hepatocellular carcinoma; HCV, hepatitis C virus; MELD, Model for End‐Stage Liver Disease; OR, odds ratio; PV, portal vein; SFSS, small‐for‐size graft syndrome; SLW, standard liver weight.

Taken together, these data demonstrate that both GW cutoff values of GW/SLW 35% for the recipients with BMI ≤ 30 kg/m^2^ and GRWR 0.7% for the recipients with BMI > 30 kg/m^2^ can be clinically used to predict SFSS after LDLT.

## 4 DISCUSSION

This is the first report to demonstrate the importance of proper use of GRWR and GW/SLW for graft selection according to recipient physique in the setting of LDLT. Taken together, the way to calculate the GW cutoff values should be changed, according to the recipient BMI value; a GRWR of 0.7% should be set as the minimum GW cutoff for the recipients with BMI > 30 kg/m^2^ and GW/SLW of 35% for the recipients with BMI ≤ 30 kg/m^2^, to prevent postoperative SFSS after LDLT.

Unlike deceased donor liver transplantation, where GW is relatively irrelevant as the graft is taken from the whole liver, securing a sufficient GW for the recipient is important in LDLT. The accepted safe and ideal cutoff value for GRWR has been reported in many institutions to be 0.7% and for GW/SLW to be 35%.^10^, ^11^, ^15^, ^26^ However, the choice of living donors is often limited, and it may not be possible to find a graft with adequate GW in all cases. In such situations, the surgeon is often faced with the difficult option of accepting a graft of low volume, raising the risk of SFSS. Another point is that with the two major methods to calculate adequate GW (GRWR and GW/SLW), no report has been published discussing which approach provides the better cutoff value. The former calculates the SLW from the height and weight of the recipient, and in the latter only the weight of the recipient is the main factor. In fact, it is also true that there is a certain degree of difference between the two calculations, even in the same recipient. From 2005, we have applied a graft meeting the GW/SLW > 35% criterion as the method to measure the minimum GW for LDLT with a favorable outcome.^4^, ^10^ However, some recipients developed postoperative SFSS even when the graft had sufficient volume with GW/SLW ≥ 35%; 16.0% of recipients (13/81) with the grafts of GW/SLW ≥ 35% but GRWR < 0.7% had SFSS after surgery. There were some factors to be addressed, such as recipient condition, Model for End‐stage Liver Disease score, donor age, and portal vein pressure^5^, ^6^, ^19^; however, we have clinically predicted that physique of the recipient is also a major factor for SFSS. Therefore, the present finding that the method for calculating the GW cutoff value should be changed according to the physique (GRWR 0.7% for recipients with BMI > 30 kg/m^2^ and GW/SLW 35% for recipients with BMI ≤ 30 kg/m^2^) has developed to be clinically useful for the recipients who undergo LDLT.

Regarding the relationship between portal hypertension and the two formulas for GW, in the recipients with BMI ≤ 30 kg/m^2^ (n = 664), of the cohort of PV pressure at closure > 20 mm Hg, the recipients with GW/SLW < 35% had a significantly higher incidence of SFSS than those with GW/SLW ≥ 35% (15.7% vs 8.2%, *P* = 0.013). Similarly, in the cohort of PV pressure ≤ 20 mm Hg, the recipients with GW/SLW < 35% had a tendency of higher incidence of SFSS than those with GW/SLW ≥ 35% (28.6% vs 21.7%, *P* = 0.603). Besides, in the recipients with BMI > 30 kg/m^2^ (n = 30), both in the cohort of PV pressure > 20 mm Hg and ≤20 mm Hg, the recipients with GRWR < 0.7% had a tendency of higher incidence of SFSS than those with GRWR ≥ 0.7% (PV pressure > 20 mm Hg, 22.2% vs 6.7%, *P* = 0.273; PV pressure ≤ 20 mm Hg, 100% vs 0%, *P* = 0.006). Collectively, each formula adds some predictability (about 7 to 16%), for SFSS according to the recipients’ physique without depending on the presence of portal hypertension; however, in the future, a new detailed analysis is needed after the accumulation of case numbers.

Graft volume and remnant liver volume are critical factors for recipient survival and donor safety, respectively.^2^, ^6^, ^7^ However, many institutions manage to select the appropriate graft in consideration of the recipient’s metabolic demands for liver regeneration postoperatively.^10^, ^11^, ^12^, ^13^, ^14^, ^15^, ^16^, ^17^ Because the metabolic needs of the recipient may be determined by the physique of the recipient and multiple additional factors, such as age of both recipient and donor, gender, status of infection, portal hypertension, presence of major collateral vessels, other organ status as well as the Model for End‐stage Liver Disease score, there is no accurate and determinant indicator to measure the metabolic needs so far. In general, the grafts with GW/SLW < 35% but GRWR ≥ 0.7% (type 2) and the grafts with GRWR < 0.7% but GW/SLW ≥ 35% (type 3) indicated the marginal donors in LDLT. Indeed, using the criteria of our hospital (GW/SLW ≥ 35%), the type 2 grafts cannot be applied; however, 18 patients underwent LDLT at another hospital where the criterion is GRWR ≥ 0.7%. However, 81 patients underwent LDLT (type 3) that cannot be performed at an institution where the criterion is GRWR ≥ 0.7% but can be performed in our hospital. From the present results, if BMI matches, the recipients who undergo LDLT using these marginal grafts are expected to have a graft that meets the metabolic demands after LDLT, with a favorable clinical course and without SFSS. This may help ameliorate the stagnation of LDLT because of donor shortages. The mechanism of the relationship between physique and metabolic demand for liver regeneration or preventing SFSS after LDLT should be elucidated in the future.

The other important issue that needs to be addressed is the increasing incidence of obese and overweight recipients in most transplant centers, even in Asia. The concern relates to the impact of obesity on the outcomes after LDLT.^28^ For the relationship between GRWR and the incidence of SFSS, Lee et al^26^ reported SFSS in three out of 23 (13%) recipients of grafts with GRWR < 0.7%, and in the study of Selzner et al,^29^ 9% of recipients with low GRWR < 0.8%, developed SFSS after LDLT. The same features were also seen in the present study where 16.0% of recipients with GRWR < 0.7% had SFSS as a consequence. However, when the recipients were limited to overweight or obese recipients with BMI > 30 kg/m^2^, a recent study of 1325 patients reported significantly increased morbidity, such as SFSS and infective complications, in overweight and obese recipients – even in deceased donor liver transplantation.^30^ Contrary to that result, Agarwal et al^31^ suggested that there was no difference in the postoperative outcomes between the two groups (n = 864); body weight ≥ 100 kg group (mean BMI, 35.8 kg/m^2^) and body weight < 100 kg group (mean BMI, 25.2 kg/m^2^). However, in this study, the mean GRWR was relatively high at 1.02% in the normal BW group and even 0.74% in the overweight group that meet our GW criteria. From the clinical standpoint in that report, that most recipients had sufficient GW, it was difficult to examine the efficacy of applying the GRWR calculation in obese recipients. However, we demonstrated that in obese recipients with BMI > 30 kg/m^2^, the recipients with GRWR ≥ 0.7% had a significantly lower incidence of SFSS at 5.9%, compared with those with GRWR < 0.7% at 46.2%. Regarding the prognosis, in the obese recipients, the recipients with GRWR < 0.7% had a significantly higher incidence of 30‐day mortality (38.5% vs 5.9%, *P* = 0.024) than those with GRWR ≥ 0.7%, and a higher tendency of 6‐month graft loss (46.2% vs 17.7%, *P* = 0.091). Furthermore, the cutoff value of GW/SLW 35% could not significantly differentiate the incidence of SFSS and short‐term prognosis. Taken together, GRWR is likely to be much more effective for the recipients who are obese with BMI > 30 mg/m^2^ in LDLT. The threshold of the minimum GW can be adjusted individually according to the physique of the recipient.

A limitation of the present study is the small number of recipients with BMI > 30 kg/m^2^ (30 patients). Therefore, further investigations will be needed.

In conclusion, proper use of the formulas for calculating GRWR and GW/SLW in choosing the graft according to recipient BMI for LDLT is very important, not only to meet the metabolic demand for liver regeneration after LDLT, but also to ameliorate the donor shortage. In addition, the mechanism of the relationship between physique and metabolic demand after LDLT warrants further research.

## DISCLOSURE

Funding: This study was supported by the following 5 grants; the Program for Basic and Clinical Research on Hepatitis, from the Japan Agency for Medical Research and Development, AMED (Numbers 20fk0210035s0503, 20fk0310106h0204, and 19fm0208009h0003); JSPS KAKENHI, a Grant‐in‐Aid from the Ministry of Health, Labour and Welfare, Japan (Numbers JP‐18K08542); and Taiju Life Social Welfare Foundation 2020. The funding sources had no role in the collection, analysis, or interpretation of the data, or in the decision to submit the article for publication.

Conflicts of Interest: The authors declare no conflicts of interest.

Author Contribution: T. Toshima participated in the writing of the manuscript. T. Yoshizumi participated in the conception and design of the study. Shimagaki, H. Wang, and T. Kurihara participated in the acquisition of the data. T. Toshima, Y. Nagao, S. Itoh, and N. Harada participated in the statistical analysis and interpretation of the data. T. Yoshizumi participated in the review of the manuscript. M. Mori participated in the review of the manuscript and final approval.

Ethical Approval: All procedures performed in studies involving human participants were in accordance with the ethical standards of the institutional review board of the ethics committee, national research committee, as well as with the 1964 Helsinki declaration and its later amendments. The study protocol was approved by the Institutional Review Board (No. 2019‐186). Informed consent was obtained from all individual participants included in the study.
